# Human-Phosphate-Binding-Protein inhibits HIV-1 gene transcription and replication

**DOI:** 10.1186/1743-422X-8-352

**Published:** 2011-07-15

**Authors:** Thomas Cherrier, Mikael Elias, Alicia Jeudy, Guillaume Gotthard, Valentin Le Douce, Houda Hallay, Patrick Masson, Andrea Janossy, Ermanno Candolfi, Olivier Rohr, Eric Chabrière, Christian Schwartz

**Affiliations:** 1 Institut de Parasitologie et Pathologie Tropicale, EA 4438, Université de Strasbourg, 3 rue Koeberlé, 67000 Strasbourg, France; 2 Laboratoire URMITE - UMR 6236 Faculté de Médecine, 27, Bvd Jean Moulin, 13385 Marseille Cedex 5 France; 3 Unité d'enzymologie, Département de Toxicologie, centre de recherche du service de santé des armées, 38702 la Tronche, France; 4 IUT Louis Pasteur de Schiltigheim, 1 Allée d'Athènes, 67300 Schiltigheim, France; 5 Institut Universitaire de France, 103 Bvd St-Michel, 75005 Paris, France; 6 Cellular and Molecular Biology Unit, FUSAGx, Gembloux, Belgium; 7 Weizmann Institute of Science, Biological Chemistry, Rehovot, Israel

**Keywords:** HIV-1, HPBP, transcription, HAART

## Abstract

The Human Phosphate-Binding protein (HPBP) is a serendipitously discovered lipoprotein that binds phosphate with high affinity. HPBP belongs to the DING protein family, involved in various biological processes like cell cycle regulation. We report that HPBP inhibits HIV-1 gene transcription and replication in T cell line, primary peripherical blood lymphocytes and primary macrophages. We show that HPBP is efficient in naïve and HIV-1 AZT-resistant strains. Our results revealed HPBP as a new and potent anti HIV molecule that inhibits transcription of the virus, which has not yet been targeted by HAART and therefore opens new strategies in the treatment of HIV infection.

## Introduction

Human immunodeficiency 1 (HIV-1), identified in 1983 [1], remains a global health threat responsible for a world-wide pandemic. The introduction of the highly active antiretroviral therapy (HAART) in 1996 exhibited the potential of curing acquired immune deficiency syndrome (AIDS). Even though an effective AIDS vaccine is still lacking, HAART has greatly extended survival [2]. AIDS pandemic has stabilized on a global scale in 2008 with an estimated 33 million people infected worldwide (data from UN, 2008).

However, several problems have been encountered since the introduction of HAART, and improvements in the design of drugs for HIV-1 are needed. A drawback of HAART is that the treatment is very expensive with limitation of its use to western countries. HAART has also several serious side effects leading to treatment interruption. Another major concern is related to the emergence of multidrug resistant viruses which has been reported in patients receiving HAART [3-5]. Therefore, new antiviral drugs are needed with activities against both wild type and mutant viruses. Two major cellular targets for HIV-1 are currently known which have critical role in HIV pathogenesis, i.e. CD4+ T lymphocytes and monocytes/macrophages including microglial cells, which are the central nervous system resident macrophages [6-8]. However, several drugs being active in CD4+ T lymphocytes are ineffective in chronically infected macrophages (i.e. several reverse transcriptase inhibitors) [9], and protease inhibitors have significantly lower activities in macrophages compared to lymphocytes [10]. Finally, many observations strongly suggest that even long term suppression of HIV-1 replication by HAART cannot totally eliminate HIV-1. The virus persists in cellular reservoirs because of viral latency, cryptic ongoing replication or poor drug penetration [11-13]. Moreover, these cellular reservoirs are often found in tissue sanctuary sites where penetration of drugs is restricted, like in the brain [14-16]. All these considerations (existence of several reservoirs, tissue-sanctuary sites and multidrug resistance) urge the search for new and original anti HIV-1 treatment strategies. Currently there are seven classes of antiretroviral (ARV) drugs available in the treatment of HIV-1-infected patients: nucleoside/nucleotide reverse transcriptase inhibitors (NRTIs), nucleotide reverse transcriptase inhibitors (NtRTIs), non-nucleoside reverse transcriptase inhibitors (NNRTIs), protease inhibitors (PIs), entry/fusion inhibitors (EIs), co-receptor inhibitors (CRIs) and integrase inhibitors (INIs) [17]. The therapy of HIV-1-infected patients is based on a combination of three or more drugs from two or more classes [18]. There have been attempts without success to develop vaccines against HIV- 1 and this field of research needs new directions [19-21]. Improvement of HAART is therefore crucial.

We believe that new drugs should target other steps of the HIV-1 cycle such as transcription since there is no drug currently available targeting this step. An increasing number of studies suggest that inhibitors of cellular LTR-binding factors, such as NF-KB and Sp1 repress LTR-driven transcription [19,21-24]. Recently, it has been shown that proteins of the DING family are good candidates to repress HIV-1 gene transcription [25,26].

More than 40 DING proteins have now been purified, mostly from eukaryotes [[27] and personal communication] and most of them are associated with biological processes and some diseases [28]. The ubiquitous presence in eukaryotes of proteins structurally and functionally related to bacterial virulence factors is intriguing, as is the absence of eukaryotic genes encoding DING proteins in databases. However, theoretical arguments together with experimental evidences supported an eukaryotic origin for DING proteins [29,30]. A member of the DING family proteins, HPBP, was serendipitously discovered in human plasma while performing structural studies on another target, the HDL-associated human paraoxonase hPON1 [31-33]. The structure topology is similar to the one described for soluble phosphate carriers of the ABC transporter family [32-36] that makes HPBP the first potential phosphate transporter identified in human plasma. Moreover, the association with hPON1 has been hypothesized to be involved in inflammation and atherosclerosis processes [37]. Later, the *ab initio *sequencing of HPBP by tandem use of mass spectrometry and X-ray crystallography confirmed that its gene was missing from the sequenced human genome [38]. Immunohistochemistry studies performed in mouse tissues demonstrate that DING proteins are present in most of tissues, spanning from neurons to muscle cells and their cellular localization is largely variable, being exclusively nuclear in neurons, or nuclear and cytoplasmic in muscle cells [30]. Altogether, these localizations are consistent with the biological function that was associated to these proteins, especially the regulation/alteration of cell cycle.

To test whether HPBP is a potential HIV-1 repressor we carried out experiments in a lymphoblastoid cell line (Jurkat) and in primary cells (Peripherial Blood Lineage and macrophage cultures). We report that HPBP represses HIV-1 replication through the inhibition of its gene transcription. Furthermore, HPBP is also active against mutant viruses. Evidence that HPBP can block HIV-1 LTR promoted expression and replication should lead to the design of new drugs which target a not yet targeted step of the virus cycle i.e. transcription.

## Materials and methods

### Protein purification

HPBP/HPON1-containing fractions were obtained following previously described HPON1 purification protocol [39]. Then HPBP was purified from these fractions according to Renault *et al. *protocol [33]. HPBP/HPON1-containing fraction in 25 mM Tris buffer containing 0.1% Triton X-100, were injected on Bio-Gel HTP hydroxyapatite (BioRad Laboratories, Munich, Germany) equilibrated with 10 mM sodium phosphate pH 7.0. This step was followed by washing with the same buffer and elution by 400 mM sodium phosphate allowed to separate the two proteins. HPBP was not retained on hydroxyapatite equilibrated without CaCl_2 _and was collected in the filtrate. On the contrary, HPON1 was retained and subsequently eluted by higher phosphate concentrations.

### Cell culture

1G5 cells (a Jurkat stable cell line for LTR-luciferase) were grown in RPMI 1640 medium supplemented with 10% fetal calf serum and in the presence of penicillin and streptomycin (100 U/ml). Primary Macrophages were cultured and prepared as previously described [40].

### Antiretroviral compounds

Stock of AZT (Glaxo Wellcome) was prepared as 0.1 mM solution in dimethylsulfoxide (Pierce) and stored at -70°C. Stock solutions were further diluted in culture medium immediately prior to use.

### Luciferase assays

1G5 cells (a Jurkat stable cell line for LTR-luciferase) were transfected (5 × 10^6^-10^7 ^cells/transfection) using DEAE-dextran transfection method with HIV-1 pNL4.3. Two days later, cells were collected and luciferase activity was determined using the Dual-GloTM Luciferase Assay System (Promega). Values correspond to an average of at least three independent experiments performed in duplicate.

### HIV-1 infection and viral replication

1G5 cells (a Jurkat stable cell line for LTR-luciferase) were transfected (5 × 10^6^-10^7 ^cells/transfection) using DEAE-dextran transfection method with HIV-1 pNL4.3. After 24 h indicated amount of HPBP was added to cell culture medium. HIV-1 replication was monitored as described previously [41].

Purified PBLs were prepared from peripheral blood of healthy donors as described previously [42]. For purified PBL preparation, Ficoll-Hypaque (Pharmacia, Uppsala, Sweden)-isolated PBMCs were incubated for 2 h on 2% gelatincoated plates. Nonadherent cells, 98% that were PBLs, as assessed by CD45/CD14 detection by flow cytometry analysis (Simultest Leucogate, Becton Dickinson, San Jose, CA, USA), were harvested after Ficoll-Hypaque isolation and adherence. PBLs were cultivated in RPMI with 10% (v/v) FBS supplemented with human recombinant IL-2 (20 IU/ml) following treatment with PHA (5 μg/ml) for 48 h. Cultured in 24-well plates, cells were electroporated (Biorad Gene Pulser X Cell) with the complete HIV-1 infectious molecular clone pNL4.3. For infection experiments, cells were infected (50 ng/million cells) with a wt lymphotropic strain pNL4.3 or an AZT resistant lymphotropic strain (purchased by NIH AIDS research and reference program (lot number 0014 A018-G910-6, post AZT isolates) [43]. HIV-1 replication was monitored as described previously [40].

Macrophages cells were cultured and prepared as previously described [40]. Cultured in 24-well plates, cells were transfected using Lipofectamine 2000 reagent (Invitrogen, Carlsbad, CA, USA) with the complete HIV-1 infectious molecular clone pNL4.3. For infection experiments, cells were infected (50 ng/million cells) with the pseudo typed pNL4.3-VSV 1 virus. Vesicular stomatitis virus G protein (VSV-G) pseudotyped virions were produced by cotransfection of 293T cells with 500 ng of VSV-G expressed with plasmid pHCMVg along with 2 μg of the proviral clone. HIV-1 replication was monitored as described previously [40]. Values correspond to an average of at least three independent experiments carried out in duplicates.

### MTT assay

Jurkat cells, as well primary cells, i.e PBL and macrophages, were seeded in 96-well plates and indicated amount of HPBP was added to cell culture medium. The possible cytotoxic effect of the antiretroviral compounds tested was examined using a 3- [4,5-Dimethylthiazol-2-yl]-2,5-diphenyltetrazolium bromide (MTT) assay [44]. Cells were grown at 37°C/5% CO2 for 6 days in the presence of antiretroviral compounds at individual concentrations of 100, 20, 5, or 0 nM, before removal of the supernatant and replacement with 0.25 mg/ml MTT (Sigma) in phenol red-free RPMI-1640 (Life Technologies). After incubation at 37°C/5% CO2 for 1 h, the MTT-containing supernatant was removed and the cells lysed with 5 ml of isopropanol:1M HCl (96:4 v/v). Triplicate 100 _l volumes of dye-containing supernatant were transferred to a 96-well ELISA-plate (Nunc) and the absorption measured at 570 nm, using background subtraction at 630 nm.

### Statistical analysis

Values are the means and SDs of independent experiments. Statistical analysis was performed by Student's *t *test, and differences were considered significant at a value of *p *< 0.05.

## Results

### 1. HPBP represses HIV-1 gene transcription and replication

**Figure 1:**
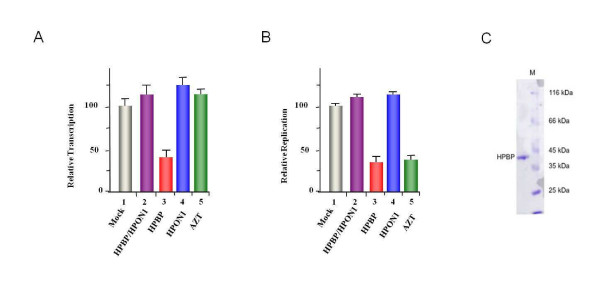
**HPBP represses HIV-1 gene transcription and replication**. 1G5 cells were transfected with the pNL4.3 provirus. (1) Mock, (2) HPBP/HPNO1 (50 nM), (3) HPBP (50 nM), (4) HPON1 (50 nM), and (5) AZT (10 μM) was added 24 h post transfection. Luciferase activity (A) and HIV-1 replication (B) were monitored 48 h post transfection. Values correspond to an average of at least three independent experiments carried out in duplicate. The purity and the size of purified-HPBP were controlled by SDS-PAGE and coomassie blue staining (C).

In order to assess the anti HIV-1 activity of HPBP, we tested HPBP, HPON1 and the complex HPBP/HPON1, for their activities on HIV-1 gene transcription and replication. The complex HPBP-HPON (Figure 1.A and 1.B lane 2) and the purified HPON1 (Figure 1.A and 1.B lane 4) did not have significant impact neither on HIV-1 replication nor on HIV-1 gene transcription. However, purified HPBP strongly repressed HIV-1 replication and transcription (respectively 60 and 70% as shown in Figure 1.A and 1.B lane 3). AZT treatment (10 μM), used as a control, was efficient to repress HIV-1 replication but not HIV-1 gene transcription (Figure 1.A and 1.B lane 5). Heat-inactivated HPBP, used in another control experiment, had no effect on HIV-1 replication (data not shown).

### 2. Dose response and cytotoxicity assay for HPBP

**Figure 2:**
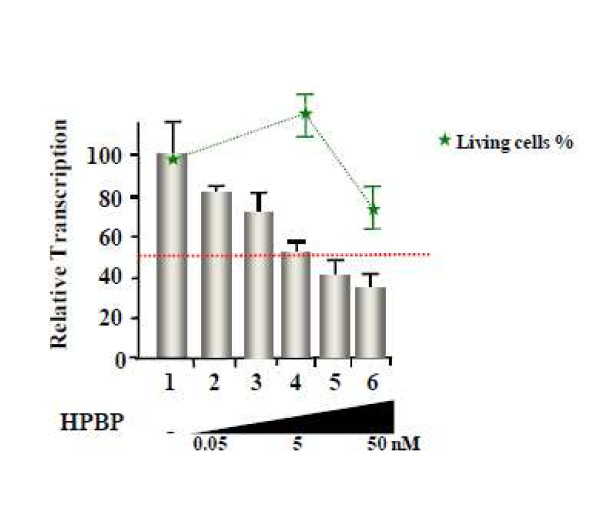
**Dose response and cytotoxicity test**. 1G5 cells were infected with NL4-3 and treated with increasing amount of HPBP 24 h post transfection. Luciferase activity was assessed 48 h post transfection. MTT test were performed in the same conditions than the dose response experiment. HIV-1 inhibition (grey columns) and percentage of living cell (green line) are shown relative to the mock treated conditions as 100%. Values correspond to an average of at least three independent experiments performed in duplicate.

Figure 2 shows the dose response effect of HPBP on the Jurkat cells with an IC50 (50% inhibitory concentration) equal to 5 nM. To measure the cytotoxic effect of HPBP on these cells we used the MTT [3-(4,5-dimethylthiazol-2-yl)-2,5-diphenyltetrazolium bromide] cytotoxicity assay [44]. Results, shown in Figure 2 (green line), allowed us to calculate CC50 (50% toxicity concentration) to be equal to 526 nM. We next performed dose response experiments and MTT cytotoxicity assays in primary cells. In Peripherical Blood Cells (PBL), the IC50 is estimated to 5 nM and the CC50 is estimated to 200 nM. Comparable results were obtained in primary macrophages with an IC50 of 5 nM and a CC50 of 140 nM (see table 1).

**Table 1:** CC50 and IC50 values in peripheral blood lymphocytes and in primary macrophages

	Peripheral Blood Lymphocytes	Primary Macrophages
CC50	200 nM	140 nM

IC50	5 nM	5 nM

### 3. HPBP is efficient against drug-resistant strain of HIV-1

**Figure 3:**
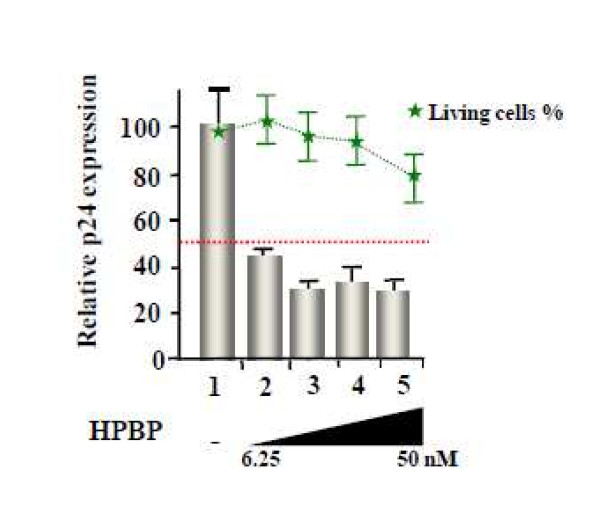
**Dose response of HPBP on an AZT-resistant HIV-1 strain**. PBL were infected with AZT resistant strain of HIV-1. Different concentrations of HPBP were added 24 h post infection and the HIV-1 replication was monitored 3 days post infection by quantification of the viral p24 protein. HIV-1 inhibition (grey columns) and percentage of living cell (green line) are shown relative to the mock treated conditions as 100%. The 100% level of replication correspond to an average 1.1 ng of p24 protein/ml. Values correspond to an average of at least three independent experiments performed in duplicate.

To assess the anti HIV-1 activity of HPBP against mutant viruses, we performed a dose response effect of HPBP on PBL infected with an HIV-1 AZT-resistant strain (lot number 0014 A018-G910-6, post AZT isolates) [43]. As shown in Figure 3, HPBP is active against the mutant strain with an IC50 (5 nM) to the same extent as observed for the wild type strain.

## Discussion

HPBP is a member of the DING protein family identified in eukaryotes for their implication in diverse biological processes [28,37]. Here, we show that the human phosphate binding protein has a potent anti HIV-1 activity. Previous observations suggested that p27^SJ^, another member of the DING protein family isolated from the plant *Hypericum perforatum*, represses HIV-1 replication and transcription [25,45,46]. However, it is noteworthy to precise that this inhibitor effect is dose dependent. Indeed it was shown by the same group that p27^SJ ^has a dual role on MCP1(monocyte chemoattractant protein 1) gene transcription being an activator at low concentration and an inhibitor at high concentration [47]. This lead us to hypothesize that HPBP might also have antiviral activities. Since CD4+ T lymphocytes and cells from monocyte-macrophage lineages are major targets for HIV-1, we assessed the *in vitro *antiviral activity of HPBP in lymphoblastoid cell lines (Jurkat), primary monocyte/macrophage cells and peripheral blood lymphocytes presenting laboratory and clinical isolates of HIV-1. The inhibitory effect of HPBP on HIV-1 replication is very strong, the IC50 value being in the range of 5 nM to 10 nM, and also compared to other canonical drugs currently used in HAART (15 nM to 6.7 μM for AZT and 40 nM to 8.5 μM for tenofivir) [48]. At this concentration HPBP is also a potent anti HIV-1 drug in PBL and in primary macrophages, which is not true for several other anti HIV-1 drugs. For example, 3 RT inhibitors, i.e. Lamivudine, entricitabine and AZT have different IC50 values when assessed for their antiviral activity in PBL and macrophages [49]. Furthermore, the CC50 values for HPBP were in the range of 140 nM to 200 nM and the selectivity index CC50/IC50 (ratio between the toxic dose and the inhibitory dose) of HPBP was in the range of 28 to 40. This high ratio indicates that the therapeutical index should therefore be high enough for use in *in vivo studies*.

HPBP also emerged as a promising candidate for drug development as it targets HIV-1 transcription, a phase of the HIV-1 cycle not yet targeted by other drugs. In productive cells, the transcription of the provirus DNA is regulated by the interplay of a combination of viral and cellular transcription factors [50-53]. Darbinian and coll. have identified the protein P27^SJ^, which belongs to the DING protein family and inhibits the activity of the HIV-1 promoter by interfering with NF-IL6, RNA pol II and Tat [45,46]. Targeting cellular factors, i.e. NF-IL6 has the advantage that resistance to these new drugs should evolve with a lower probability. More importantly, interfering with Tat will ensure a strong and selective repression of HIV-1 replication.

Since its introduction in 1996, antiretroviral therapy has changed the clinical course of HIV and AIDS. However drug resistance has occurred with all of the antiretroviral agents. It is now a major public health concern and it is crucial to design new antiretroviral drugs [54,55]. These new drugs should inhibit HIV replication by targeting new steps within the viral life cycle. Of great interest HPBP, which targets transcription, is as effective against drug resistant HIV strains as to wild type strains, highlighting the potential therapeutic advantage of HPBP. The molecular mechanism of action is unknown but currently under investigation. In the future, pharmacophores ("part of a molecule that is necessary to ensure the optimal interactions with a specific biological target and to trigger (or block) its biological response") can be inferred from the characterization of these biochemical studies.

In conclusion, this work indicates that HPBP has a potent anti HIV activity through the inhibition of transcription a not yet targeted phase of the virus cycle. However additional experiments regarding the HPBP impact on HIV replication and gene transcription have to be performed on other viral strains including several other mutant strains (NRTIs, NNRTIs and protease inhibitors). We believe that this protein or its derivatives are potentially interesting molecules and deserve further studies. As suggested for X-DING-CD4 [26], this work could also uncover a new function for proteins belonging to the DING protein family, that is a role in the innate response to infection including HIV-1. New investigations will be needed in order to precise the importance of the DING proteins. It has been previously shown that HPBP is tightly associated with HPON1 [56]. The search of a correlation between the HPBP abundance, its biologic availability, the HPBP/HPON ratio and the non progression in the disease AIDS in the "elite non progressors cohort" will be of great interest. Finally DING proteins may constitute a marker for AIDS progression since it has been shown that both HPON activity and its concentration have been altered in the presence of HIV-1 [57,58].

## Competing interests

The authors declare that they have no competing interests.

## Authors' contributions

TC, ME, Alicia J, carried out dose response and cytotoxicity assays in lymphocytes and macrophages. Andrea J participated in dose response and cytotoxicity assays in macrophages. VLD and HH carried experiments with heat-inactivated HPBP. CS carried out experiments in Jurkat cell line. GG and PM purified HPBP. Ermanno C and OR participated in the design of the study. Eric C and CS conceived of the study, participated in its design and coordination and wrote the paper. All authors read and approved the final manuscript.
